# Crystal structure of bis­(1-ethyl­pyridinium) dioxonium hexa­cyanidoferrate(II)

**DOI:** 10.1107/S2056989017000810

**Published:** 2017-01-20

**Authors:** Rikako Tanaka, Nobuyuki Matsushita

**Affiliations:** a Department of Chemistry and Research Center for Smart Molecules, Rikkyo University, Nishi-Ikebukuro 3-34-1, Toshima-ku, 171-8501 Tokyo, Japan

**Keywords:** crystal structure, mol­ecular salt, oxonium ion, hexa­cyanidoferrate(II), ethyl­pyridinium ion, hydrogen bonds

## Abstract

The title compound, (C_7_H_10_N)_2_(H_3_O)_2_[Fe(CN)_6_] or (Etpy)_2_(H_3_O)_2_[Fe(CN)_6_] (Etpy^+^ is 1-ethyl­pyridinium), crystallizes in the space group *Pnnm*. The Fe^II^ atom of the [Fe(CN)_6_]^4−^ anion lies on a site with site symmetry ..2/*m*, and has an octa­hedral coordination sphere defined by six cyanido ligands. Both the Etpy^+^ and the oxonium cations are located on a mirror plane. In the crystal, electron-donor anions of [Fe(CN)_6_]^4−^ and electron-acceptor cations of Etpy^+^ are each stacked parallel to the *b* axis, resulting in a columnar structure with segregated moieties. The crystal packing is stabilized by a three-dimensional O—H⋯N hydrogen-bonding network between the oxonium ions and the cyanide ligands of [Fe(CN)_6_]^4−^.

## Abstract 2

The crystal structure of the title compound comprises a segregated columnar structure of cations and anions extending parallel to the *b* axis

## Chemical context   

Prussian blue is a well-known compound which displays a deep-blue colour based on an inter­valence charge-transfer inter­action between [Fe^II^(CN)_6_]^4−^ electron-donor species and Fe^III^ electron-acceptor species. Several charge-transfer salts composed of [Fe(CN)_6_]^4−^ and organic acceptor cations, *e.g.* 1,1′-dimethyl-4,4′-bipyridinium (methyl viologen) have been reported (Nakahara & Wang, 1963 ▸; Kostina *et al.*, 2001 ▸; Kotov *et al.*, 2005 ▸; Abouelwafa *et al.*, 2010 ▸). In the majority of cases, the reported charge-transfer salts of [Fe(CN)_6_]^4−^ are accompanied by dicationic organic acceptor species. On the other hand, charge-transfer salts of [Fe(CN)_6_]^4−^ accompanied by monocationic species are rather rare (Gorelsky *et al.*, 2007 ▸).
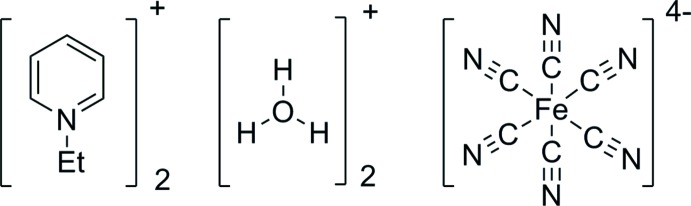


The present X-ray crystallographic analysis of the title salt, (Etpy)_2_(H_3_O)_2_[Fe(CN)_6_] (Etpy^+^ is 1-ethyl­pyridinium), (I), was performed in order to elucidate the crystal packing of a charge-transfer hexa­cyanidoferrate(II) anion with a monocationic organic acceptor and an oxonium ion.

## Structural commentary   

**Figure 1:**
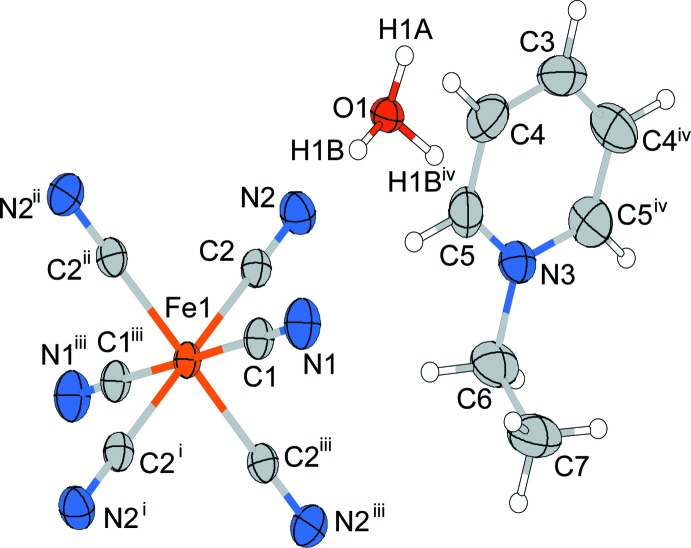
The structures of the mol­ecular components of compound (I), showing the atomic numbering scheme. Displacement ellipsoids are drawn at the 50% probability level for non-H atoms. [Symmetry codes: (i) −*x*, −*y*, −*z*; (ii) *x*, *y*, −*z*; (iii) −*x*, −*y*, *z*; (iv) *x*, *y*, −*z* + 1.]

The structures of the mol­ecular components of (I) are displayed in Fig. 1 ▸. The asymmetric unit of (I) contains half of an Etpy^+^ cation and an oxonium ion (both located on a mirror plane), and one quarter of an [Fe(CN)_6_]^4−^ anion, the Fe^II^ atom of which is located on a site with symmetry ..2/*m*. The Fe^II^ atom is coordinated by six cyanido ligands in a slightly distorted octa­hedral configuration [Fe—C = 1.9045 (18), 1.9068 (13) Å; C≡N = 1.157 (2), 1.1598 (17) Å; C—Fe—C_*trans*_ = 180.0°; C—Fe—C_*cis*_ = 89.60 (7)–90.40 (7)°; Fe—C—N = 178.67 (18), 179.77 (13)°]. The bond angle of the ethyl group of the Etpy^+^ ion [N3—C6—C7 = 110.77 (19) °] is similar to those of Etpy[AlCl_4_] [109.2 (11)°; Zaworotko *et al.*, 1989 ▸] and poly[4-di­methyl­amino-1-ethyl­pyridin-1-ium [tri-*μ*-dicyanamido-*κ* ^6^ *N* ^1^:*N* ^5^-cadmium]] [111.5 (5)°; Wang *et al.*, 2015 ▸].

## Supra­molecular features   

**Figure 2:**
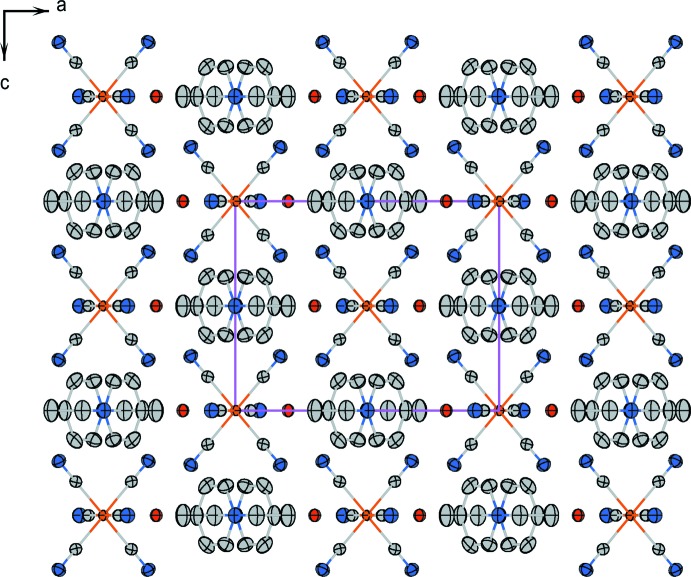
The crystal packing of compound (I) in a view along the *b* axis. H atoms have been omitted for clarify; the probability function is as in Fig. 1 ▸.

The projection of the crystal structure of (I) along the *b* axis is shown in Fig. 2 ▸.

The [Fe(CN)_6_]^4−^ electron-donor anions and the Etpy^+^ electron-acceptor cations stack separately in columns parallel to the *b* axis whereby both types of columns are alternately arranged in the *a-* and *c*-axis directions.

**Figure 3:**
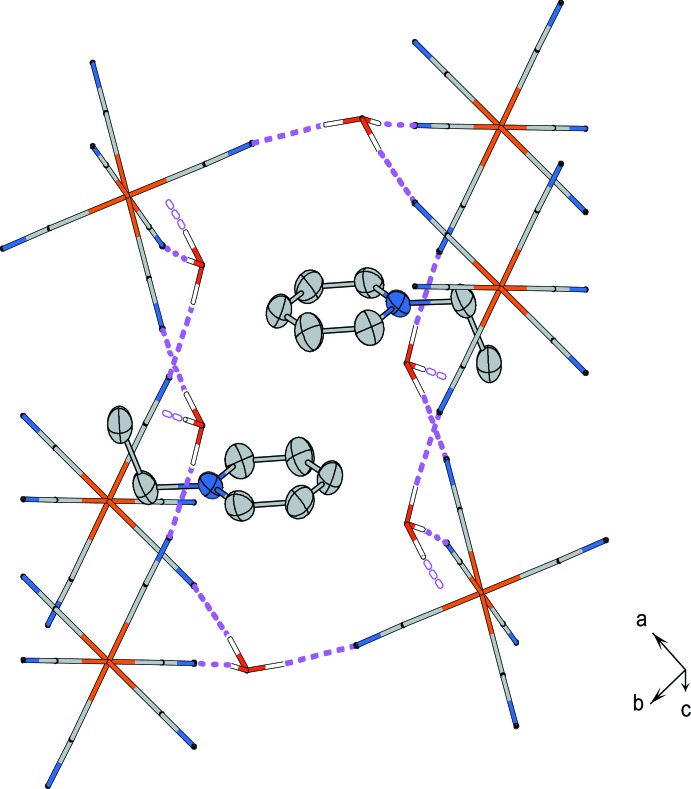
Hydrogen-bonding network composed of [Fe(CN)_6_]^4−^ anions and oxonium cations. Magenta dashed lines represent hydrogen bonds.

In the crystal of (I), the oxonium ions and [Fe(CN)_6_]^4−^ ions form a three-dimensional O—H⋯N hydrogen-bonding network (Table 1 ▸). A pair of Etpy^+^ cations is enclosed in the hydrogen-bonding cage formed by six [Fe(CN)_6_]^4−^ ions and six oxonium ions (Fig. 3 ▸). Two pyridinium rings of the Etpy^+^ cations are arranged in parallel and the ethyl groups are alternating with each other. The centroid-to-centroid distance (4.147 Å) and the face-to-face distance of the least-square planes (3.731 Å) between two pyridinium rings indicate that π–π inter­actions are not developed.

## Database survey   

Several crystal structures of compounds containing the Etpy^+^ cation have been deposited in the Cambridge Structural Database (Groom *et al.* 2016 ▸), *e.g.* Etpy[AlCl_4_] (Zaworotko *et al.*, 1989 ▸), Etpy[Ni(mnt)_2_] (mnt = maleo­nitrile-1,2-di­thiol­ate; Robertson *et al.*, 1999 ▸), or (Etpy)_2_[CoCl_4_] (Felloni *et al.*, 2004 ▸). A hexa­cyanidoferrate(II) salt, (Hpy)_2_(H_3_O)_2_[Fe(CN)_6_] (Hpy^+^ = *N*-hydro­pyridinium; Gorelsky *et al.*, 2007 ▸), quite similar to (I), has been also reported.

## Synthesis and crystallization   

**Figure 4:**
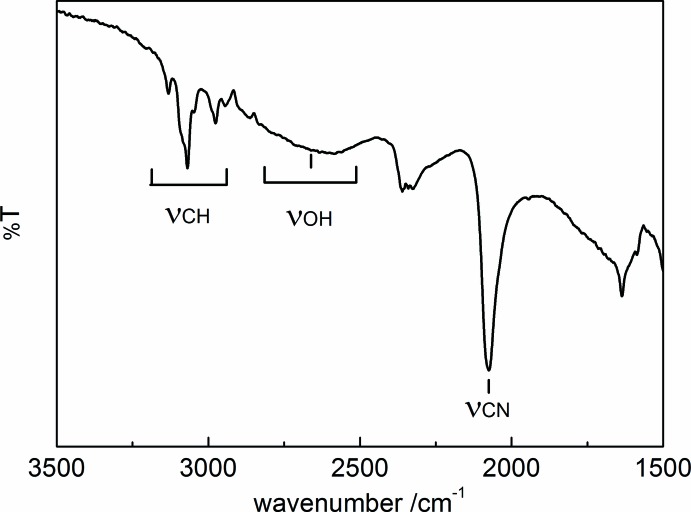
The IR spectrum of compound (I).

H_4_[Fe(CN)_6_] (106 mg) and l-ascorbic acid (60 mg) were dissolved in water (17 ml). The mixture was added to an aqueous solution of 1-ethyl­pyridinium bromide (177 mg/17 ml). After standing at 277 K for a day, yellow platelet-shaped crystals suitable for X-ray analysis were obtained. Elemental analysis: found: C, 51.52; H, 5.878; N, 24.06%; calculated for C_20_H_26_FeN_8_O_2_: C, 51.51; H, 5.63; N, 24.03%. Thermogravimetry was measured from 296 to 476 K at a rate of 5 K min^−1^ under N_2_ gas flow (100 ml min^−1^) on a Rigaku TG-DTA Thermo Plus EVO2 TG8121. Found: 7.85% mass loss; calculated: 7.73%. The mass loss of (I) took place at around 373 to 393 K and corresponds to two water mol­ecules per chemical formula. The result suggests that the water mol­ecules are released from the oxonium ions. Most probably, protons, H^+^, remain in the crystal as counter-cations. The IR spectrum of compound (I) is shown in Fig. 4 ▸. Selected IR bands (KBr pellet, cm^−1^): 3135–2941 (*s*, C—H, *str*), 2640 (*br*, O—H, *str*), 2075 (*s*, C≡N, *str*).

## Refinement   

Crystal data, data collection and structure refinement details are summarized in Table 2 ▸. In the final refinement of the title compound, three reflections, *viz*. (0 17 1), (2 16 0) and (5 15 2), were omitted owing to poor agreements between observed and calculated intensities. H atoms of the Etpy^+^ cation were, at first, located in a difference Fourier map, but finally placed in geometrically calculated positions and refined as riding, with C(methyl­ene)—H = 0.92 Å, C(meth­yl)—H = 0.98 Å and C(aromatic)—H = 0.95 Å, all with *U*_iso_(H) = 1.5*U*_eq_(C). H atoms of the oxonium ion were located in a difference Fourier map and their positions refined with *U*_iso_(H) = 1.5*U*_eq_(O). The maximum and minimum electron density peaks are located 1.00 Å from atom C1 and 0.71 Å from atom Fe1, respectively.

## Supplementary Material

Crystal structure: contains datablock(s) I. DOI: 10.1107/S2056989017000810/wm5355sup1.cif

Structure factors: contains datablock(s) I. DOI: 10.1107/S2056989017000810/wm5355Isup2.hkl

CCDC reference: 1527928

Additional supporting information:  crystallographic information; 3D view; checkCIF report

## Figures and Tables

**Table 1:** Hydrogen-bond geometry (Å, °)

*D*—H⋯*A*	*D*—H	H⋯*A*	*D*⋯*A*	*D*—H⋯*A*
O1—H1*A*⋯N1^i^	0.90 (3)	1.67 (3)	2.569 (2)	176 (3)
O1—H1*B*⋯N2	0.931 (18)	1.632 (19)	2.5589 (15)	173.6 (18)

Symmetry code: (i) 
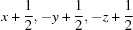
.

**Table 2:** Experimental details

Crystal data	
Chemical formula	(C_7_H_10_N)_2_(H_3_O)_2_[Fe(CN)_6_]
*M*_r_	466.34
Crystal system, space group	Orthorhombic, *P* *n* *n* *m*
Temperature (K)	173
*a*, *b*, *c* (Å)	11.8807 (4), 12.1279 (7), 8.3962 (2)
*V* (Å^3^)	1209.79 (9)
*Z*	2
Radiation type	Mo *K*α
μ (mm^−1^)	0.65
Crystal size (mm)	0.28 × 0.13 × 0.08

Data collection	
Diffractometer	Rigaku R-AXIS RAPID imaging-plate
Absorption correction	Multi-scan (*ABSCOR*; Higashi, 1995 ▸)
*T*_min_, *T*_max_	0.907, 0.952
No. of measured, independent and observed [*I* > 2σ(*I*)] reflections	25575, 2216, 1840
*R*_int_	0.034
(sin θ/λ)_max_ (Å^−1^)	0.746

Refinement	
*R*[*F* ^2^ > 2σ(*F* ^2^)], *wR*(*F* ^2^), *S*	0.036, 0.093, 1.11
No. of reflections	2213
No. of parameters	88
H-atom treatment	H atoms treated by a mixture of independent and constrained refinement
Δρ_max_, Δρ_min_ (e Å^−3^)	0.50, −0.70

Computer programs: *RAPID-AUTO* (Rigaku, 2006 ▸), *SHELXT2014* (Sheldrick, 2015*a* ▸), *SHELXL2014* (Sheldrick, 2015*b* ▸), *DIAMOND* (Brandenburg, 2016 ▸) and *publCIF* (Westrip, 2010 ▸).

## supplementary crystallographic information

### Crystal data

**Table:**

(C_7_H_10_N)_2_(H_3_O)_2_[Fe(CN)_6_]	*D*_x_ = 1.280 Mg m^−^^3^
*M**_r_* = 466.34	Mo *K*α radiation, λ = 0.7107 Å
Orthorhombic, *P**n**n**m*	Cell parameters from 8639 reflections
*a* = 11.8807 (4) Å	θ = 3.4–32.0°
*b* = 12.1279 (7) Å	µ = 0.65 mm^−^^1^
*c* = 8.3962 (2) Å	*T* = 173 K
*V* = 1209.79 (9) Å^3^	Block, pale-yellow
*Z* = 2	0.28 × 0.13 × 0.08 mm
*F*(000) = 488	

### Data collection

**Table:**

Rigaku R-AXIS RAPID imaging-plate diffractometer	2216 independent reflections
Radiation source: X-ray sealed tube	1840 reflections with *I* > 2σ(*I*)
Graphite monochromator	*R*_int_ = 0.034
Detector resolution: 10.00 pixels mm^-1^	θ_max_ = 32.0°, θ_min_ = 3.4°
ω scans	*h* = −17→17
Absorption correction: multi-scan (*ABSCOR*; Higashi, 1995)	*k* = −18→18
*T*_min_ = 0.907, *T*_max_ = 0.952	*l* = −12→10
25575 measured reflections	

### Refinement

**Table:**

Refinement on *F*^2^	Primary atom site location: structure-invariant direct methods
Least-squares matrix: full	Secondary atom site location: difference Fourier map
*R*[*F*^2^ > 2σ(*F*^2^)] = 0.036	Hydrogen site location: difference Fourier map
*wR*(*F*^2^) = 0.093	H atoms treated by a mixture of independent and constrained refinement
*S* = 1.11	*w* = 1/[σ^2^(*F*_o_^2^) + (0.0356*P*)^2^ + 0.6061*P*] where *P* = (*F*_o_^2^ + 2*F*_c_^2^)/3
2213 reflections	(Δ/σ)_max_ < 0.001
88 parameters	Δρ_max_ = 0.50 e Å^−^^3^
0 restraints	Δρ_min_ = −0.70 e Å^−^^3^

### Special details

**Table:**

Geometry. All esds (except the esd in the dihedral angle between two l.s. planes) are estimated using the full covariance matrix. The cell esds are taken into account individually in the estimation of esds in distances, angles and torsion angles; correlations between esds in cell parameters are only used when they are defined by crystal symmetry. An approximate (isotropic) treatment of cell esds is used for estimating esds involving l.s. planes.

### Fractional atomic coordinates and isotropic or equivalent isotropic displacement parameters (Å^2^)

**Table:**

	*x*	*y*	*z*	*U*_iso_*/*U*_eq_	
Fe1	0.0000	0.0000	0.0000	0.02095 (10)	
C1	−0.05743 (16)	0.14661 (15)	0.0000	0.0257 (3)	
C2	0.10536 (11)	0.04026 (10)	0.16115 (15)	0.0255 (2)	
N1	−0.09019 (17)	0.23642 (15)	0.0000	0.0371 (4)	
N2	0.16963 (11)	0.06441 (11)	0.25910 (15)	0.0352 (3)	
N3	0.00181 (15)	0.25836 (14)	0.5000	0.0333 (3)	
C3	0.1469 (2)	0.4327 (2)	0.5000	0.0485 (6)	
H3	0.1978	0.4930	0.5000	0.073*	
C4	0.10987 (15)	0.38888 (16)	0.3591 (2)	0.0498 (4)	
H4	0.1344	0.4192	0.2607	0.075*	
C5	0.03717 (15)	0.30083 (14)	0.3608 (2)	0.0431 (4)	
H5	0.0118	0.2698	0.2633	0.065*	
C6	−0.0794 (2)	0.16567 (19)	0.5000	0.0485 (6)	
H6A	−0.0670	0.1193	0.5954	0.073*	
C7	−0.1981 (2)	0.2077 (2)	0.5000	0.0570 (7)	
H7A	−0.2503	0.1452	0.5000	0.086*	
H7B	−0.2108	0.2527	0.5953	0.086*	
O1	0.30049 (12)	0.08078 (12)	0.5000	0.0279 (3)	
H1A	0.342 (2)	0.143 (2)	0.5000	0.042*	
H1B	0.2513 (15)	0.0800 (15)	0.414 (2)	0.042*	

### Atomic displacement parameters (Å^2^)

**Table:**

	*U*^11^	*U*^22^	*U*^33^	*U*^12^	*U*^13^	*U*^23^
Fe1	0.02418 (17)	0.02126 (16)	0.01740 (15)	0.00227 (12)	0.000	0.000
C1	0.0302 (8)	0.0250 (8)	0.0218 (7)	0.0048 (6)	0.000	0.000
C2	0.0299 (6)	0.0246 (5)	0.0220 (5)	0.0015 (4)	0.0015 (5)	−0.0001 (4)
N1	0.0454 (10)	0.0307 (8)	0.0353 (9)	0.0104 (7)	0.000	0.000
N2	0.0383 (6)	0.0393 (6)	0.0280 (5)	−0.0029 (5)	−0.0044 (5)	−0.0030 (5)
N3	0.0364 (8)	0.0254 (7)	0.0380 (9)	0.0019 (6)	0.000	0.000
C3	0.0341 (11)	0.0473 (13)	0.0641 (16)	−0.0065 (10)	0.000	0.000
C4	0.0518 (9)	0.0524 (9)	0.0451 (9)	−0.0064 (8)	0.0172 (8)	0.0013 (8)
C5	0.0522 (9)	0.0434 (8)	0.0336 (7)	−0.0007 (7)	0.0058 (7)	−0.0083 (7)
C6	0.0506 (13)	0.0276 (10)	0.0674 (17)	−0.0047 (9)	0.000	0.000
C7	0.0469 (14)	0.0416 (13)	0.083 (2)	−0.0110 (11)	0.000	0.000
O1	0.0292 (6)	0.0287 (6)	0.0257 (6)	−0.0063 (5)	0.000	0.000

### Geometric parameters (Å, º)

**Table:**

Fe1—C1^i^	1.9045 (18)	C3—C4^iv^	1.369 (2)
Fe1—C1	1.9045 (18)	C3—H3	0.9500
Fe1—C2^i^	1.9068 (13)	C4—C5	1.373 (2)
Fe1—C2^ii^	1.9068 (13)	C4—H4	0.9500
Fe1—C2^iii^	1.9068 (13)	C5—H5	0.9500
Fe1—C2	1.9069 (13)	C6—C7	1.499 (4)
C1—N1	1.157 (2)	C6—H6A	0.9900
C2—N2	1.1598 (17)	C7—H7A	0.9800
N3—C5^iv^	1.3447 (19)	C7—H7B	0.9800
N3—C5	1.3447 (19)	O1—H1A	0.90 (3)
N3—C6	1.482 (3)	O1—H1B	0.931 (18)
C3—C4	1.369 (2)	O1—H1B^iv^	0.931 (18)
			
C1^i^—Fe1—C1	180.0	C5—N3—C6	119.61 (10)
C1^i^—Fe1—C2^i^	89.78 (5)	C4—C3—C4^iv^	119.5 (2)
C1—Fe1—C2^i^	90.22 (5)	C4—C3—H3	120.2
C1^i^—Fe1—C2^ii^	90.22 (5)	C4^iv^—C3—H3	120.2
C1—Fe1—C2^ii^	89.78 (5)	C3—C4—C5	119.65 (17)
C2^i^—Fe1—C2^ii^	89.60 (7)	C3—C4—H4	120.2
C1^i^—Fe1—C2^iii^	89.78 (5)	C5—C4—H4	120.2
C1—Fe1—C2^iii^	90.22 (5)	N3—C5—C4	120.20 (16)
C2^i^—Fe1—C2^iii^	90.40 (7)	N3—C5—H5	119.9
C2^ii^—Fe1—C2^iii^	180.00 (11)	C4—C5—H5	119.9
C1^i^—Fe1—C2	90.22 (5)	N3—C6—C7	110.77 (19)
C1—Fe1—C2	89.78 (5)	N3—C6—H6A	109.5
C2^i^—Fe1—C2	180.0	C7—C6—H6A	109.5
C2^ii^—Fe1—C2	90.40 (7)	C6—C7—H7A	109.4
C2^iii^—Fe1—C2	89.60 (7)	C6—C7—H7B	109.5
N1—C1—Fe1	178.67 (18)	H7A—C7—H7B	109.5
N2—C2—Fe1	179.77 (13)	H1A—O1—H1B	110.5 (14)
C5^iv^—N3—C5	120.8 (2)	H1A—O1—H1B^iv^	110.5 (14)
C5^iv^—N3—C6	119.61 (10)	H1B—O1—H1B^iv^	102 (2)

Symmetry codes: (i) −*x*, −*y*, −*z*; (ii) *x*, *y*, −*z*; (iii) −*x*, −*y*, *z*; (iv) *x*, *y*, −*z*+1.

### Hydrogen-bond geometry (Å, º)

**Table:**

*D*—H···*A*	*D*—H	H···*A*	*D*···*A*	*D*—H···*A*
O1—H1*A*···N1^v^	0.90 (3)	1.67 (3)	2.569 (2)	176 (3)
O1—H1*B*···N2	0.931 (18)	1.632 (19)	2.5589 (15)	173.6 (18)

Symmetry code: (v) *x*+1/2, −*y*+1/2, −*z*+1/2.
